# CT-based simulation of impingement-free mechanical hip motion after unilateral total hip arthroplasty

**DOI:** 10.1186/s12891-026-10311-3

**Published:** 2026-07-31

**Authors:** Bando Kazuma, Nakamura Junichi, Hagiwara Shigeo, Kawarai Yuya, Ohtori Seiji, Orita Sumihisa

**Affiliations:** 1 Center for Frontier Medical Engineering, Chiba University, 1-33 Yayoi-cho, CFME room#B201, Inage-ku, Chiba, 263-8522 Japan; 2 Department of Orthopedic Surgery, Graduate School of Medicine, Chiba University, 1-8-1 Inohana, Chuo-ku, Chiba city, 260-0856 Japan; 3 Department of Orthopedic Surgery, Toho University Sakura Medical Center, 564-1 Shimoshizu, Sakura, 285-8741 Japan

**Keywords:** Range of motion, Leg length, Total hip arthroplasty, CT-based three-dimensional simulation

## Abstract

**Background:**

Range of motion (ROM) after total hip arthroplasty (THA) is influenced by implant-related parameters such as femoral head diameter, neck length, and stem offset. However, the quantitative effects of these parameters on ROM, leg length discrepancy (LLD), and femoral offset discrepancy remain unclear. This study aimed to quantify the effects of implant-related parameters on simulated impingement-free mechanical ROM after unilateral THA.

**Methods:**

A computed tomography (CT) -based three-dimensional (3D) simulation was performed in 100 patients who underwent unilateral THA with a clinically and radiographically healthy contralateral hip. The contralateral healthy hip was also evaluated as a patient-specific comparator. Implant configurations were simulated by varying femoral head diameter, neck length, and stem offset. Femoral head diameters of 36 mm and 32 mm, neck lengths from − 3.5 mm to + 7 mm, and standard-offset and lateral-offset stems were evaluated. Simulated impingement-free mechanical ROM was assessed in maximum flexion, maximum extension, internal rotation at 90° of flexion, and external rotation at 10° of extension. Simulated LLD and femoral offset discrepancy relative to the contralateral healthy hip were also quantified.

**Results:**

Increasing neck length improved simulated mechanical ROM in all evaluated directions, with median increases of 6.3° in flexion and 5.3° in extension for every 3.5-mm increment. Lateral offset stems further increased flexion by 16.3° compared with standard stems. Femoral head enlargement from 32 mm to 36 mm modestly improved simulated mechanical ROM, increasing flexion by approximately 2.5°. However, neck lengthening increased LLD by a median of 2.3 mm per 3.5-mm increment. The contralateral healthy hips showed median simulated ROM values of 119° in flexion, 66° in extension, 31° in internal rotation, and 48° in external rotation.

**Conclusions:**

Increasing neck length and lateral offset improved simulated impingement-free mechanical ROM after unilateral THA, whereas neck lengthening increased LLD. Femoral head enlargement also improved simulated mechanical ROM, but its effect was limited compared with those of neck length and stem offset. The contralateral healthy hip may provide contextual patient-specific information for interpreting simulated mechanical ROM in unilateral THA.

## Background

Total hip arthroplasty (THA) aims not only to prevent dislocation but also to restore physiological hip function. In contemporary practice, surgeons are required to balance two competing goals: maximizing postoperative range of motion (ROM) and accurately restoring leg length and femoral offset [1, 2]. While malposition of the acetabular or femoral components remains a major risk factor for impingement and instability, implant-related parameters such as femoral head diameter, neck length, and stem offset also substantially influence mechanical ROM before impingement occurs [3, 4].

Normal hip ROM has been evaluated in several previous studies; however, the reported values vary widely according to population characteristics and measurement methods. Using maximum hip flexion as an example, previous studies have reported values ranging from approximately 96° to 141° in asymptomatic or healthy subjects [5–10]. Conventional goniometric studies have often reported hip flexion values of approximately 120° or greater, whereas imaging-based or dynamically confirmed measurements may yield lower values because they can better distinguish true intra-articular hip motion from pelvic or lumbosacral motion [5–10]. These findings indicate that normal hip ROM is not a fixed universal value but depends on measurement technique, endpoint definition, age, sex, and individual anatomy.

The concept of impingement-free ROM has been increasingly used to evaluate prosthetic hip kinematics using computed tomography (CT)-based three-dimensional (3D) simulation [11]. Renkawitz et al. [12] demonstrated that anatomical variations alter impingement-free ROM calculations after THA, indicating that postoperative motion cannot be predicted solely by implant positioning. These findings highlight the importance of evaluating mechanical ROM under standardized alignment conditions while accounting for patient anatomy and implant geometry. Previous simulation studies have demonstrated that larger femoral heads and increased neck length improve impingement-free ROM [13]. However, excessive neck lengthening may simultaneously increase leg length discrepancy (LLD), which has been associated with altered gait mechanics and patient dissatisfaction when exceeding 5 mm [14, 15]. Despite this recognized trade-off, quantitative data describing how much simulated mechanical ROM increases per incremental change in neck length—and how these changes simultaneously affect LLD—remain limited [16, 17].

Unilateral THA provides an opportunity to use the contralateral healthy hip as a within-patient comparator. Because hip ROM shows substantial interindividual variability, comparison with population-based normative values alone may be insufficient for interpreting whether simulated postoperative mechanical ROM is appropriate for an individual patient. In unilateral hip disease, the asymptomatic contralateral hip may provide a patient-specific comparator while minimizing interindividual differences in age, sex, body size, pelvic morphology, and spinopelvic characteristics [18–20]. Therefore, the purpose of this study was to quantify the effects of femoral head diameter, neck length, and stem offset on simulated impingement-free mechanical ROM, LLD, and femoral offset discrepancy in unilateral THA using CT-based 3D simulation.

## Methods

### Study design and patient selection

This was a CT-based three-dimensional radiographic simulation study of consecutive patients who underwent unilateral THA. All procedures involving human participants were approved by the Institutional Review Board and were conducted in accordance with the Declaration of Helsinki and its later amendments. Written informed consent was obtained from all patients.

From April 2019 to March 2022, a total of 357 primary THAs performed using the direct anterior approach with a traction table were consecutively registered at our institution [21, 22]. The inclusion criteria were as follows: (1) no history of hip surgery; (2) unilateral THA with a healthy contralateral hip; and (3) implantation of an SQRUMAG+^®^ cup with Aquara VE^®^ flat polyethylene (Kyocera Corporation, Kyoto, Japan), a ZTA^®^ femoral head, and a MIRFY^®^ femoral stem (Calm Rana, Chiba, Japan; Surgical Alliance, Tokyo, Japan; and Mizuho, Tokyo, Japan). The MIRFY stem is a Zweymüller-type short stem that has been evaluated using three-dimensional CT-based methods [23]. The contralateral hip was used as a patient-specific comparator in patients with unilateral hip disease. This approach was based on previous studies in which the contralateral hip was used as an internal reference for hip morphology, motion, or implant planning in unilateral hip disease or unilateral THA [24–27]. In the present study, the contralateral hip was defined as healthy only when there was no hip pain, acetabular dysplasia, osteonecrosis of the femoral head, rheumatoid arthritis, previous hip surgery, or other hip pathology.

**Fig. 1:**
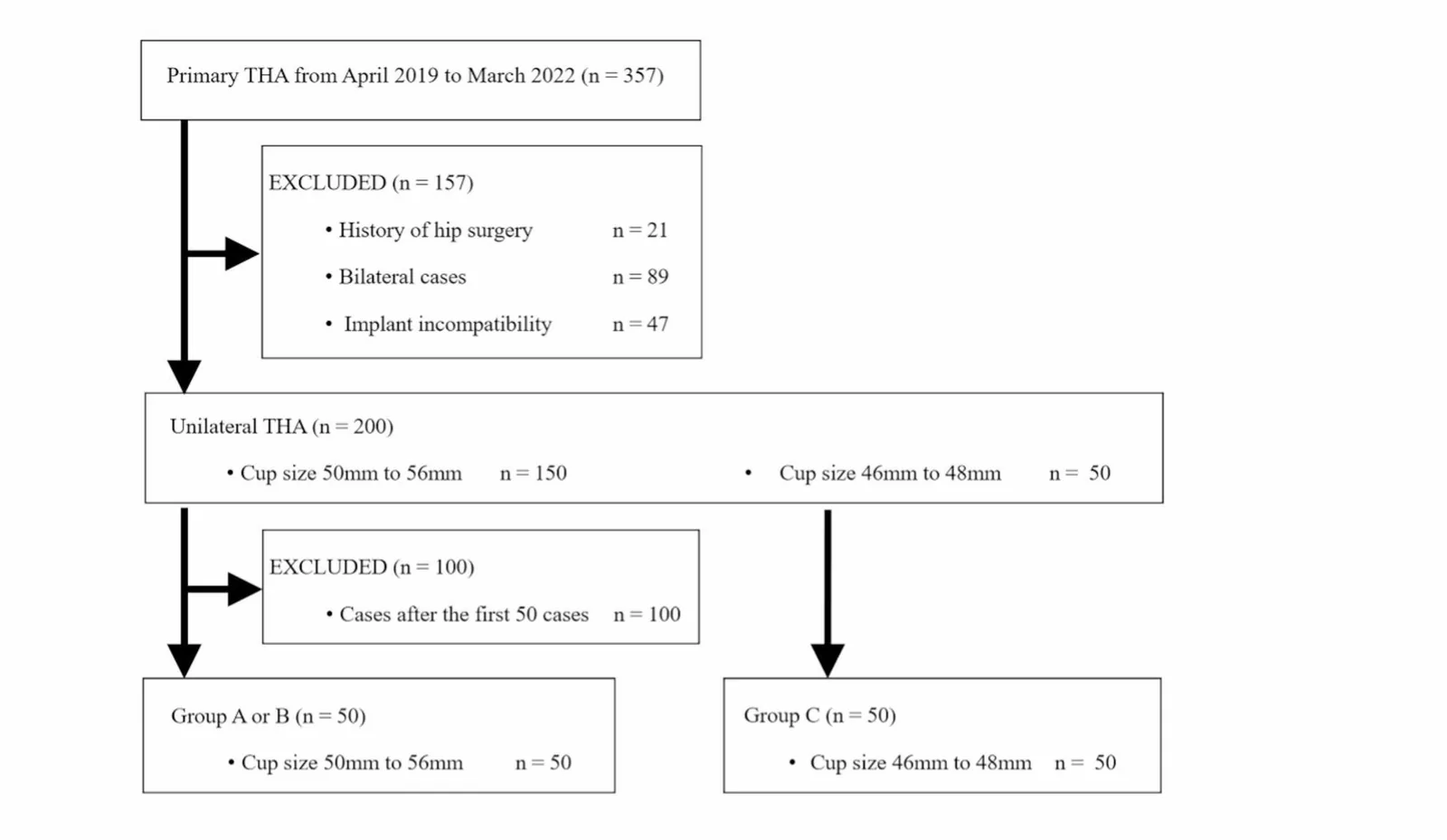
Patient selection flow diagram. Patient selection and group allocation are shown. Groups A and B included the same 50 hips with a cup size ≥50 mm and were simulated with 36-mm and 32-mm femoral heads, respectively. Group C included 50 hips with a cup size of 46 or 48 mm and was simulated with a 32-mm head

Among the 357 cases, 200 unilateral THA cases satisfied the inclusion criteria. In clinical practice, femoral head size is determined according to the cup size, which is based on the anteroposterior diameter of the acetabulum. With the SQRUMAG+^®^ system, cups ≥ 46 mm can be combined with a 32-mm femoral head, whereas cups ≥ 50 mm can be combined with a 36-mm femoral head. Using ZedHip 3D planning software (Lexi, Tokyo, Japan), the first consecutive 50 cases in which a cup ≥ 50 mm was considered suitable based on the anteroposterior acetabular diameter were selected. Similarly, the first consecutive 50 cases in which a 46-mm or 48-mm cup was deemed suitable were selected. Cases with cups ≥ 50 mm paired with a 36-mm head were classified as Group A, whereas those paired with a 32-mm head were classified as Group B. Cases with a 46-mm or 48-mm cup paired with a 32-mm head were classified as Group C. The contralateral healthy hips were used as patient-specific comparators for simulated ROM. A flow diagram of the patient selection process is shown in Fig. 1, and patient characteristics are summarized in Table 1. As indicators of femoral morphology, the canal flare index [28] and cortical index [29] were measured. Because the proportion of women in Group C was 100%, patients in this group had a shorter mean height, lower body weight, greater stem anteversion, and a narrower medullary canal compared with those in Groups A and B.

**Table 1:** Patient characteristics

	Groups A or B (*n* = 50)	Group C (*n* = 50)	*p*
Sex (male: female)	18: 32 (36%: 64%)	0: 50 (0%: 100%)	0.001
Age (years)	68.4 ± 9.9	67.5 ± 12.4	0.560
Diagnosis (OA: ONFH: RA: trauma)	38:7:3:2	38:10:1:1	0.601
Height (cm)	158.4 ± 9.2	150.6 ± 5.9	**0.001**
Weight (kg)	61.0 ± 12.8	50.4 ± 12.0	**0.003**
BMI	23.3 ± 4.0	22.1 ± 4.9	0.234
Anterior pelvic tilt angle (°)	6.2 ± 8.1	6.7 ± 5.9	0.664
Stem anteversion (°)	17.6 ± 20.5	23.0 ± 23.7	**0.039**
Canal Flare Index	3.6 ± 0.7	4.0 ± 0.6	**0.012**
Cortical Index	0.52 ± 0.07	0.54 ± 0.08	0.072
Dorr Classification (A: B: C)	30:16:4	36:12:2	0.410
Crowe Classification (1: 2: 3: 4)	46:4:0:0	44:4:2:0	0.360
Cup size, distribution: cases	50 mm: 34	46 mm: 15	**0.001**
	52 mm: 9	48 mm: 35	
	54 mm: 5		
	56 mm: 2		
Head size	36 mm (Group A)	32 mm	
	32 mm (Group B)		
Stem size	#1: 1	#01: 1	**0.001**
	#2: 7	#0: 1	
	#3: 11	#1: 16	
	#4: 15	#2: 11	
	#5: 9	#3: 12	
	#6: 5	#4: 8	
	#7: 1	#5: 1	
	#8: 1		

Mean ± standard deviation. Continuous variables were analyzed with Mann–Whitney *U* tests, and categorical variables were analyzed with Pearson χ^2^ tests. Significant differences are in bold font

*OA* osteoarthritis, *ONFH* idiopathic osteonecrosis of the femoral head, *RA* rheumatoid arthritis, *BMI* body mass index

### 3D simulation of hip range of motion

**Fig. 2:**
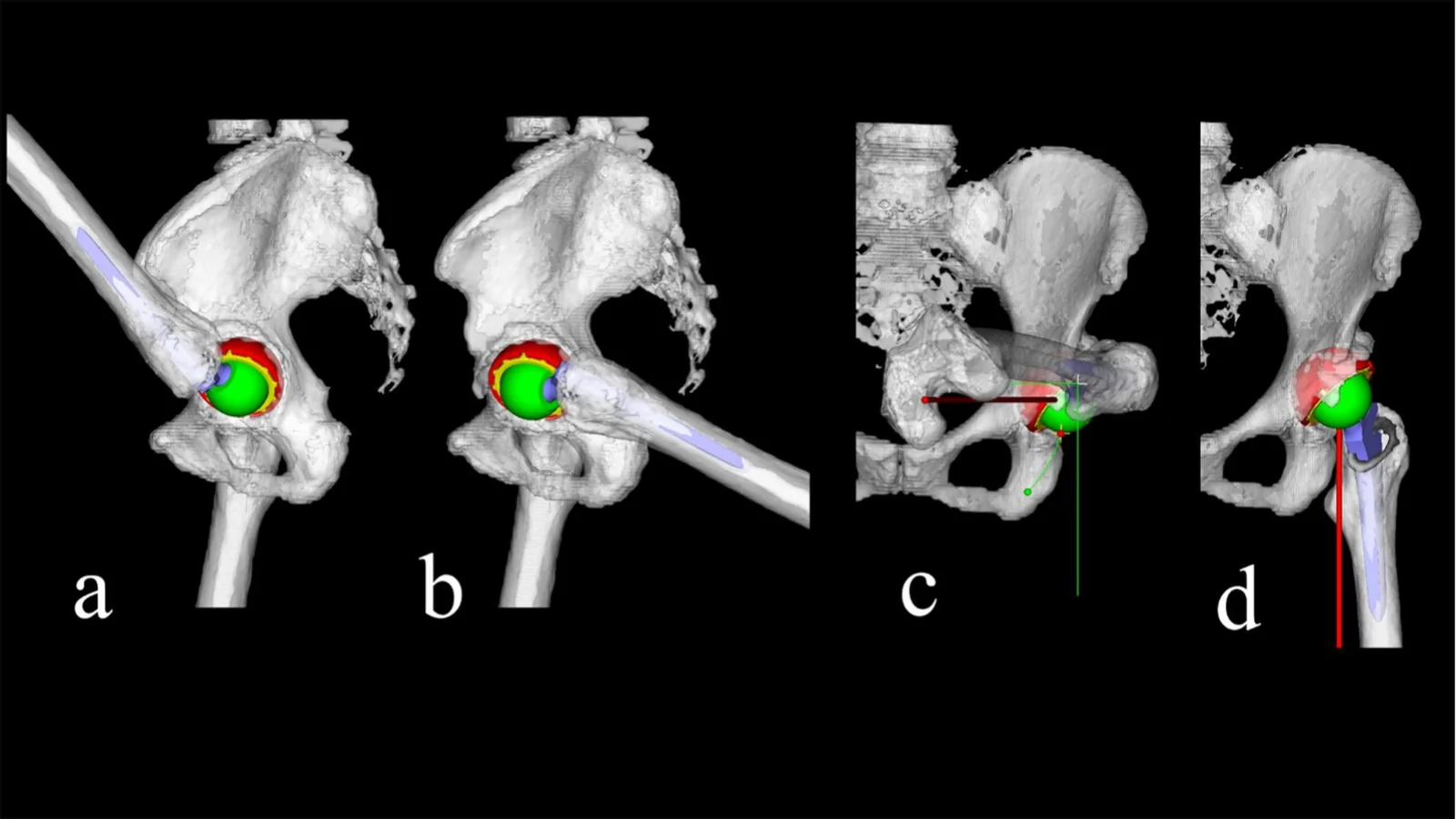
Three-dimensional simulation images at the moment of impingement. Representative images showing the simulated endpoint of impingement in the left hip: **a** maximum flexion, **b** maximum extension, **c** maximum internal rotation at 90° of flexion, and **d** maximum external rotation at 10° of extension

Preoperative CT scans from the bilateral iliac wings to the tibial tuberosities were obtained with a slice thickness of 1 mm using a helical CT scanner (Aquilion One; Toshiba Medical Systems, Tokyo, Japan). CT data were imported into ZedHip software. The reliability and validity of the ZedHip system have been previously reported, with intraclass correlation coefficients ranging from 0.907 to 0.972 [30, 31]. The functional pelvic plane was used as the pelvic coordinate system. The acetabular cup was basically placed at the anatomical acetabulum with the aim of reconstructing the anatomical hip center. In dysplastic hips, slight superior placement was allowed only when needed to obtain sufficient bony coverage, while avoiding lateralization of the hip center. Cup medialization was determined by considering the medial acetabular wall and bony coverage, while avoiding excessive medialization. Cup alignment was fixed at 40° inclination and 15° anteversion according to Lewinnek’s safe zone [32]. This standardized alignment was used to isolate the effects of implant-related parameters on simulated mechanical ROM, rather than to determine patient-specific optimal cup orientation. Stem alignment was set in a neutral position relative to the femoral axis, with stem anteversion parallel to the anatomical neck axis. Stem insertion depth was determined by considering restoration of the native femoral head center with a neutral head length. After the reference stem position was determined in each case, the stem position was kept unchanged, and only neck length and stem offset were varied. ROM, LLD, and femoral offset discrepancy were compared under these different implant conditions. Implant size was selected to best match bone morphology. Simulated impingement-free mechanical ROM was defined as the maximum hip motion before impingement between the pelvis, femur, and prosthetic components under the predefined simulation conditions. This simulated ROM represents a theoretical mechanical endpoint and does not include soft tissue constraints such as the labrum, capsule, tendons, muscle tension, or pain. ROM was simulated by varying neck length and switching between standard offset (STD) and lateral offset (LAT) stems. Simulated impingement-free mechanical ROM was evaluated in four directions: (a) maximum flexion, (b) maximum extension, (c) maximum internal rotation at 90° of flexion and 0° of abduction/adduction, corresponding to a posterior dislocation position, and (d) maximum external rotation at 10° of extension and 0° of abduction/adduction, corresponding to an anterior dislocation position (Fig. 2).

### 3D simulation of leg length and offset differences

**Fig. 3:**
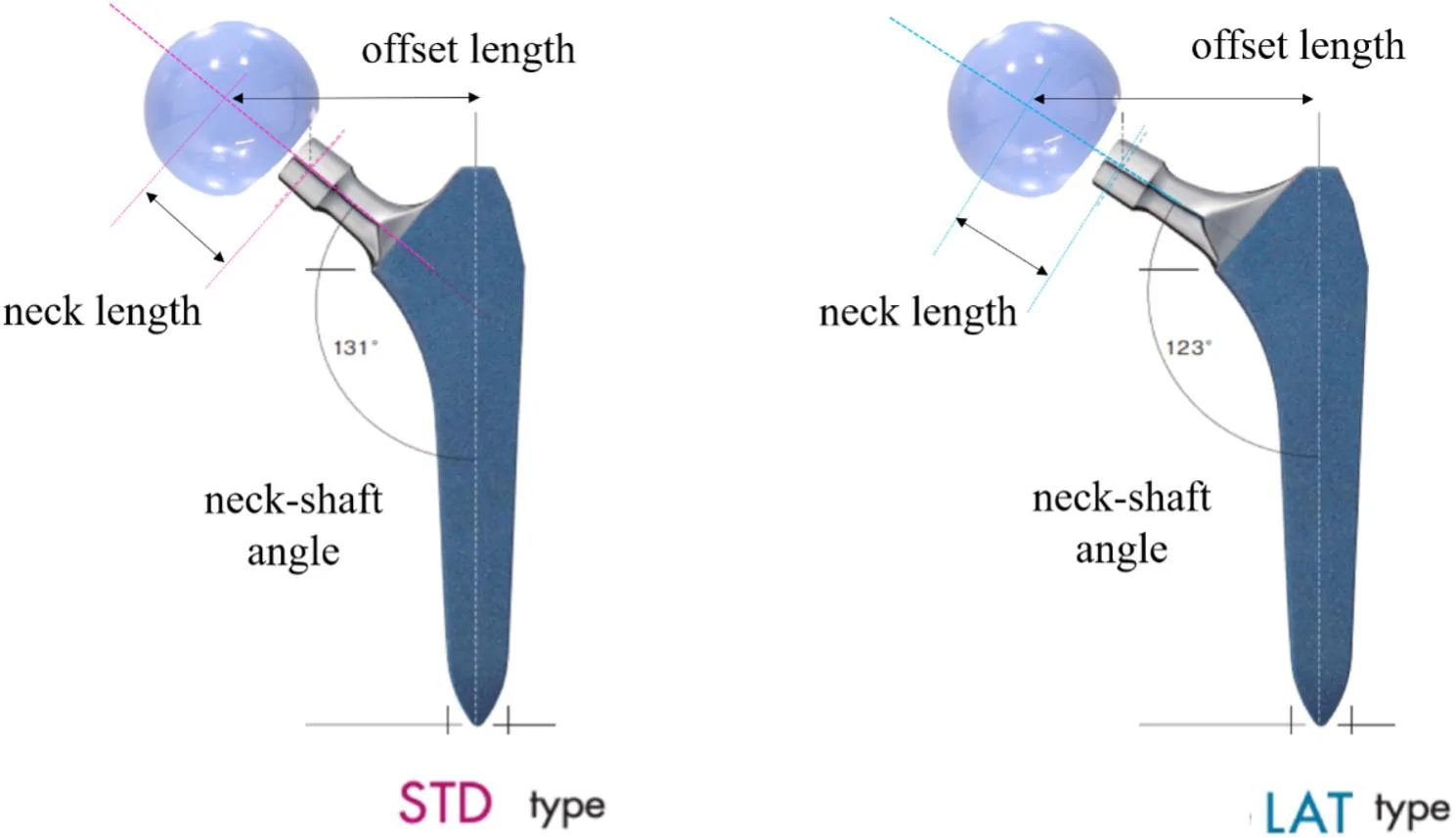
STD and LAT types of the Zweymüller short stem. Schematic illustration of the MIRFY stems. The neck–shaft angle is 131° in the STD and 123° in the LAT. Because neck length is adjusted along the femoral neck axis, changes in neck length affect both vertical height and femoral offset. The LAT stem increases femoral offset while maintaining the same femoral head center height for an identical stem size

The ZedHip system was also used to simulate LLD and femoral offset differences relative to the contralateral healthy hip. Simulations were performed by varying neck length and switching from STD to LAT stems. Neck length was adjusted to four levels: −3.5 mm, 0 mm, + 3.5 mm, and + 7 mm. The MIRFY STD stem has a neck–shaft angle of 131°, whereas the LAT stem has a neck–shaft angle of 123° (Fig. 3). The MIRFY stem is designed so that when the neck length is set to 0 mm, the shoulder of the stem aligns with the center of the femoral head. For both STD and LAT stems, the femoral head center remains at the same height when the stem size is identical. However, the offset length increases by 6.5 mm for size #1, 6 mm for size #2, and 7 mm for sizes #3–#12.

### Statistical analysis

Statistical analyses were performed using JMP Pro 15 (SAS Institute, Cary, NC, USA). Continuous variables between two independent groups were compared using the Mann–Whitney U test, whereas paired comparisons were analyzed using the Wilcoxon signed-rank test. Comparisons involving three or more groups were adjusted using the Bonferroni correction. Categorical variables were analyzed using the Pearson χ² test. A *P* value < 0.05 was considered statistically significant.

## Results

### Effects of neck length and offset on simulated impingement-free mechanical ROM in group A

**Fig. 4:**
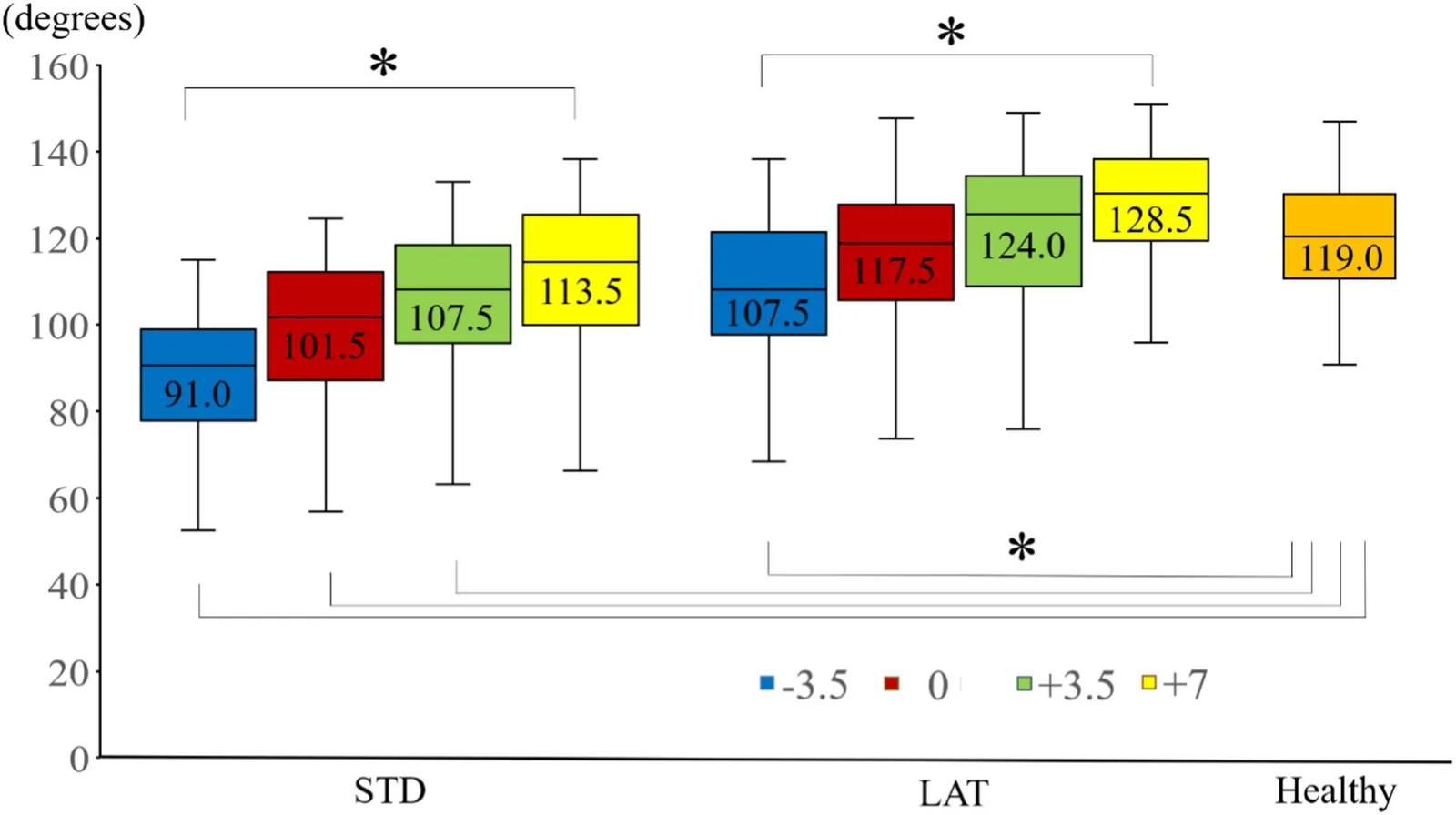
Effect of neck length and offset on simulated maximum flexion. Box plots showing simulated impingement-free mechanical flexion according to neck length and offset in hips reconstructed with a cup ≥50 mm and a 36-mm femoral head (Group A). Flexion increased with each 3.5-mm increase in neck length in both STD and LAT stems (*p* = 0.001 for each). At the same neck length, flexion was greater with the LAT stem than with the STD stem (*p* = 0.001 for each comparison). Compared with the contralateral healthy hip, flexion was smaller with STD −3.5 mm, STD 0 mm, STD +3.5 mm, and LAT −3.5 mm (*p* = 0.001, 0.001, 0.002, and 0.018, respectively)

**Fig. 5:**
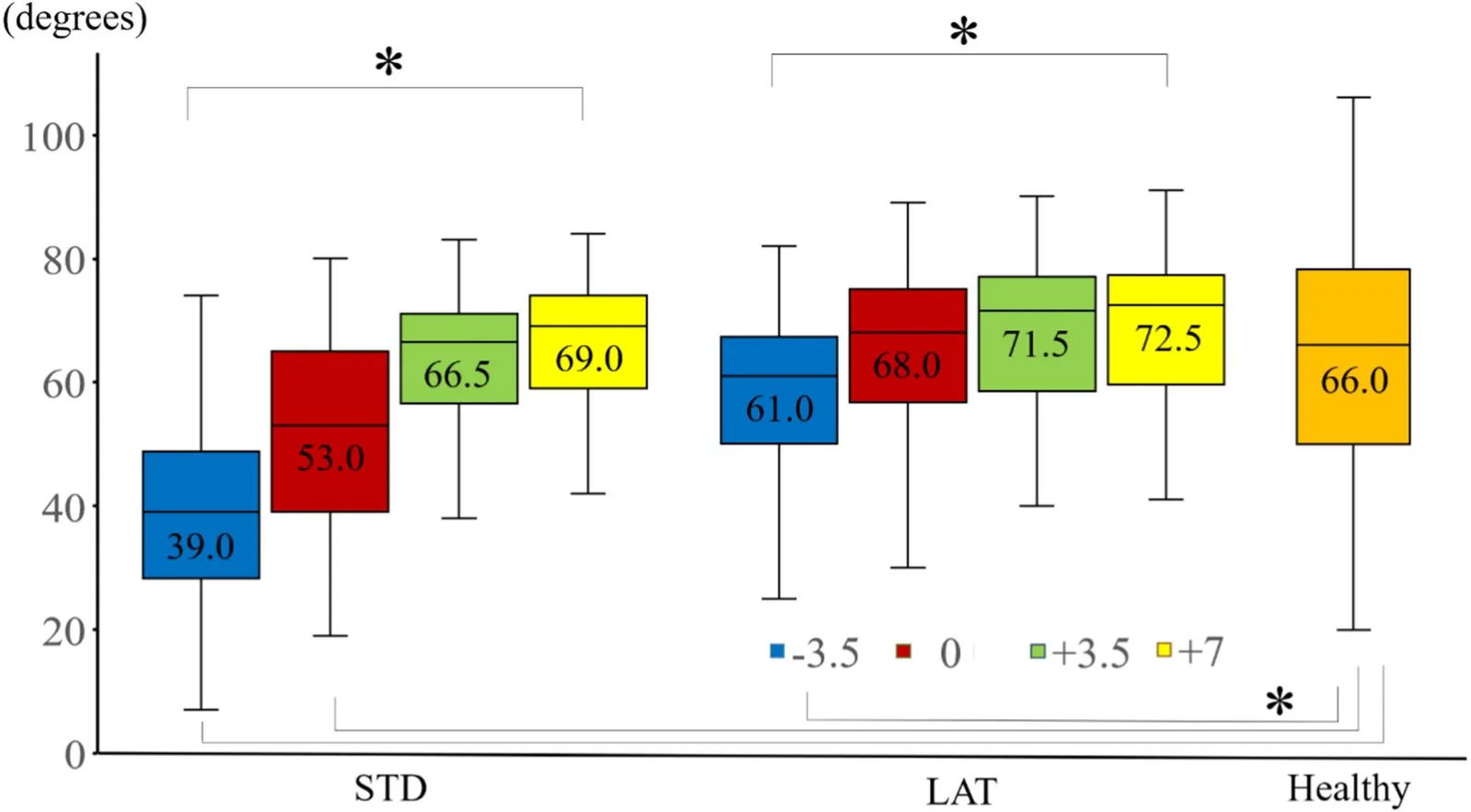
Effect of neck length and offset on simulated maximum extension. In Group A, extension increased with each 3.5-mm increase in neck length in both STD and LAT stems (*p* = 0.001 for each). At the same neck length, extension was greater with the LAT stem than with the STD stem (*p* = 0.001 for each comparison). Compared with the contralateral healthy hip, extension was smaller with STD −3.5 mm, STD 0 mm, and LAT −3.5 mm (*p* = 0.001, 0.001, and 0.002, respectively)

In Group A, increasing neck length consistently increased simulated impingement-free mechanical ROM in all evaluated directions. Maximum flexion increased by a median of 6.3° (range, 4.5°–10.0°) for every 3.5-mm increase in neck length in both STD and LAT stems (Fig. 4). At the same neck length, LAT stems increased maximum flexion by a median of 16.3° (range, 15.0°–17.5°) compared with STD stems. Maximum extension also increased by a median increase of 5.3° (range, 1.0°–14.0°) for every 3.5-mm increase in neck length (Fig. 5). At the same neck length, LAT stems produced a median of 10.0° (range, 3.5°–22.0°) greater extension than STD stems.

**Fig. 6:**
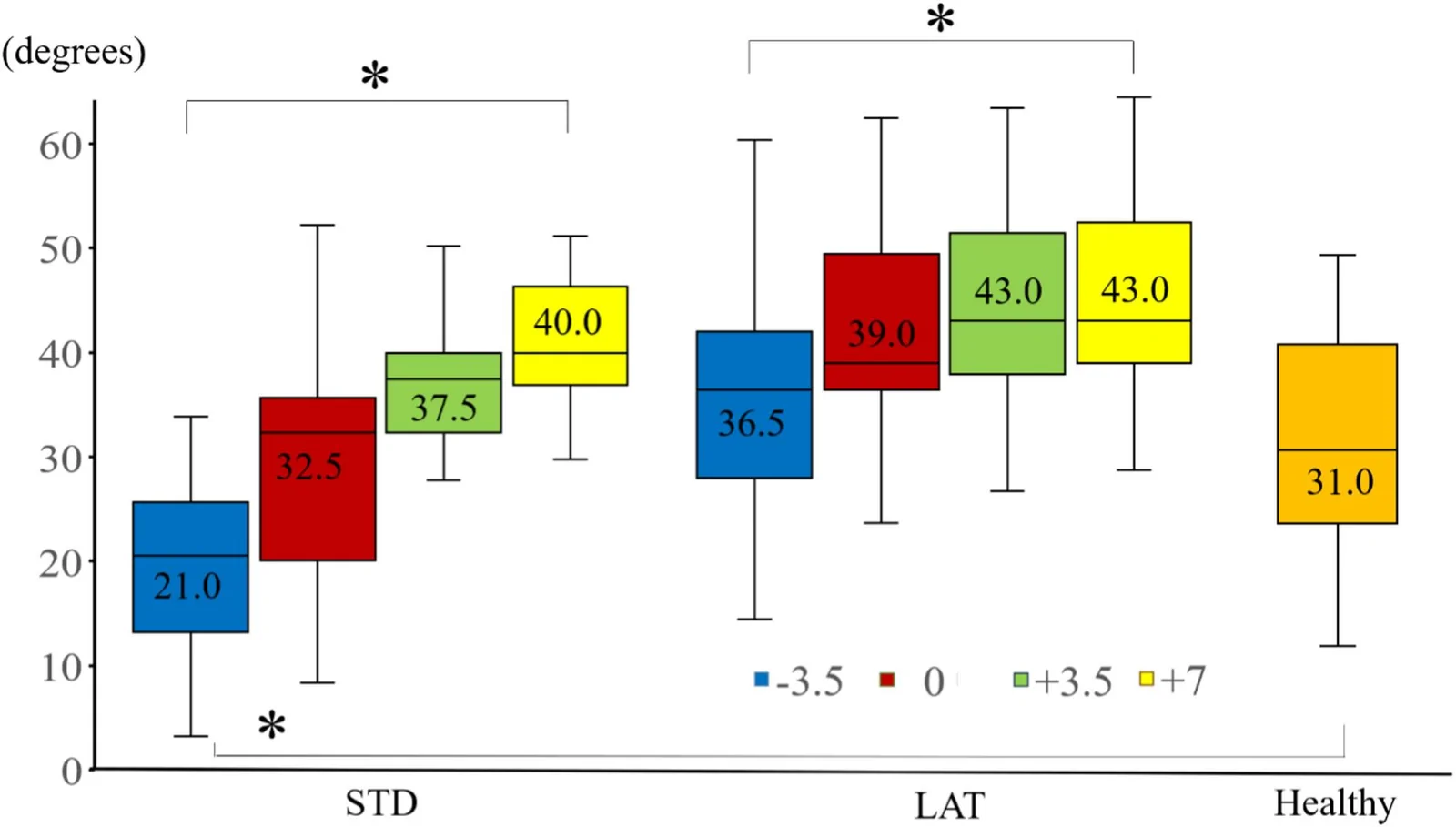
Effect of neck length and offset on simulated maximum internal rotation at 90° of flexion. In Group A, internal rotation increased with each 3.5-mm increase in neck length in both STD and LAT stems (*p* = 0.001 for each). At the same neck length, internal rotation was greater with the LAT stem than with the STD stem (*p* = 0.001 for each comparison). Compared with the contralateral healthy hip, internal rotation was smaller with STD −3.5 mm (*p* = 0.002)

**Fig. 7:**
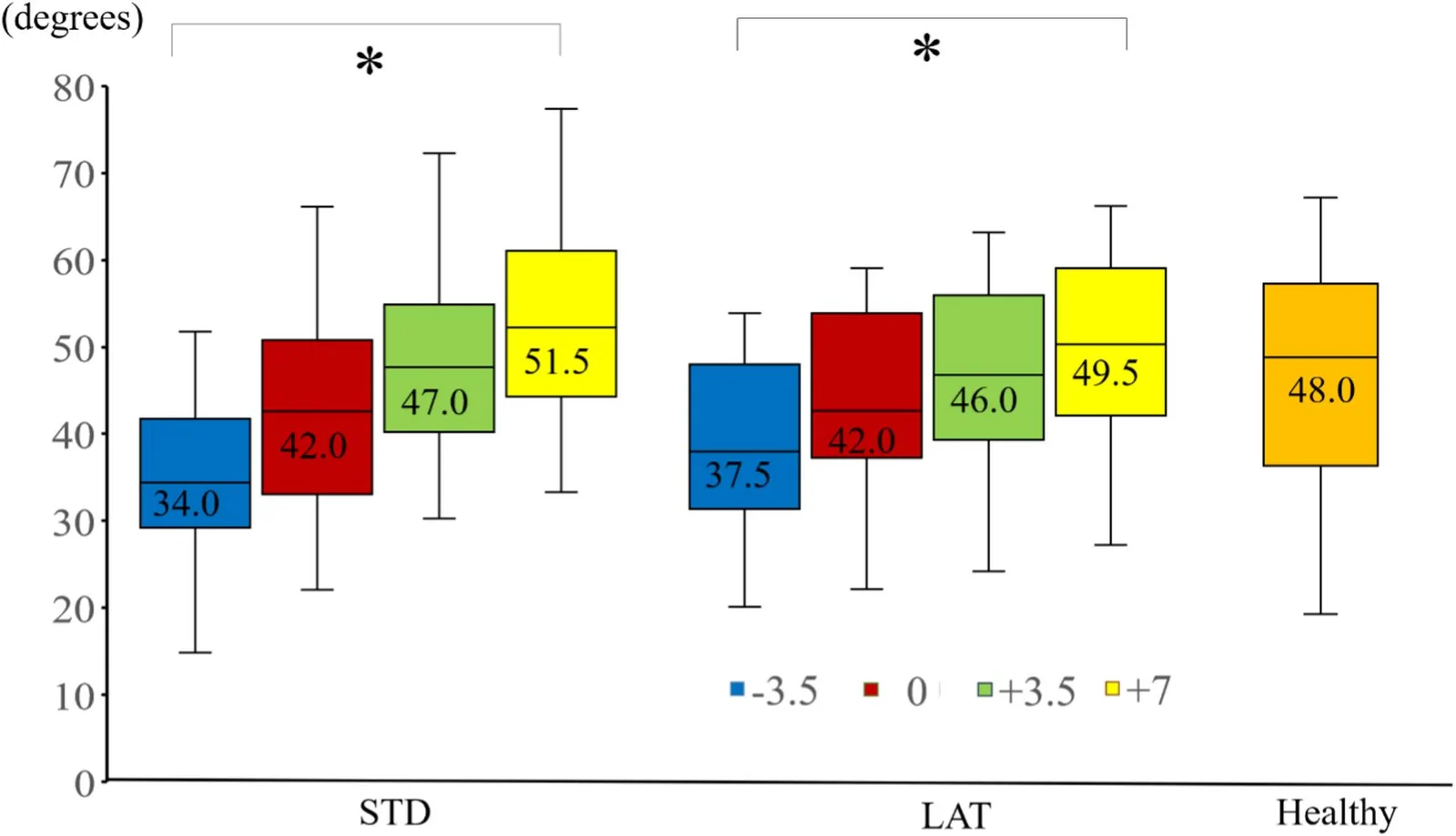
Effect of neck length and offset on simulated maximum external rotation at 10° of extension. In Group A, external rotation increased with each 3.5-mm increase in neck length in both STD and LAT stems (*p* = 0.001 for each). At the same neck length, external rotation was greater with LAT −3.5 mm than with STD −3.5 mm, but smaller with LAT +7 mm than with STD +7 mm (*p* = 0.001 for both comparisons). Compared with the contralateral healthy hip, external rotation was smaller with STD −3.5 mm and LAT −3.5 mm (*p* = 0.001 for both)

Among the 18 hips that allowed 90° of flexion, maximum internal rotation increased by a median of 3.3° (range, 0°–11.5°) for every 3.5-mm increase in neck length (Fig. 6). LAT stems further increased internal rotation by a median of 6.0° (range, 3.0°–15.5°) compared with STD stems. Among the 36 hips that allowed 10° of extension, maximum external rotation increased by a median of 3.3° (range, 1.5°–6.5°) for every 3.5-mm increase in neck length (Fig. 7).

### Effects of neck length and offset on leg length and femoral offset discrepancies

**Fig. 8:**
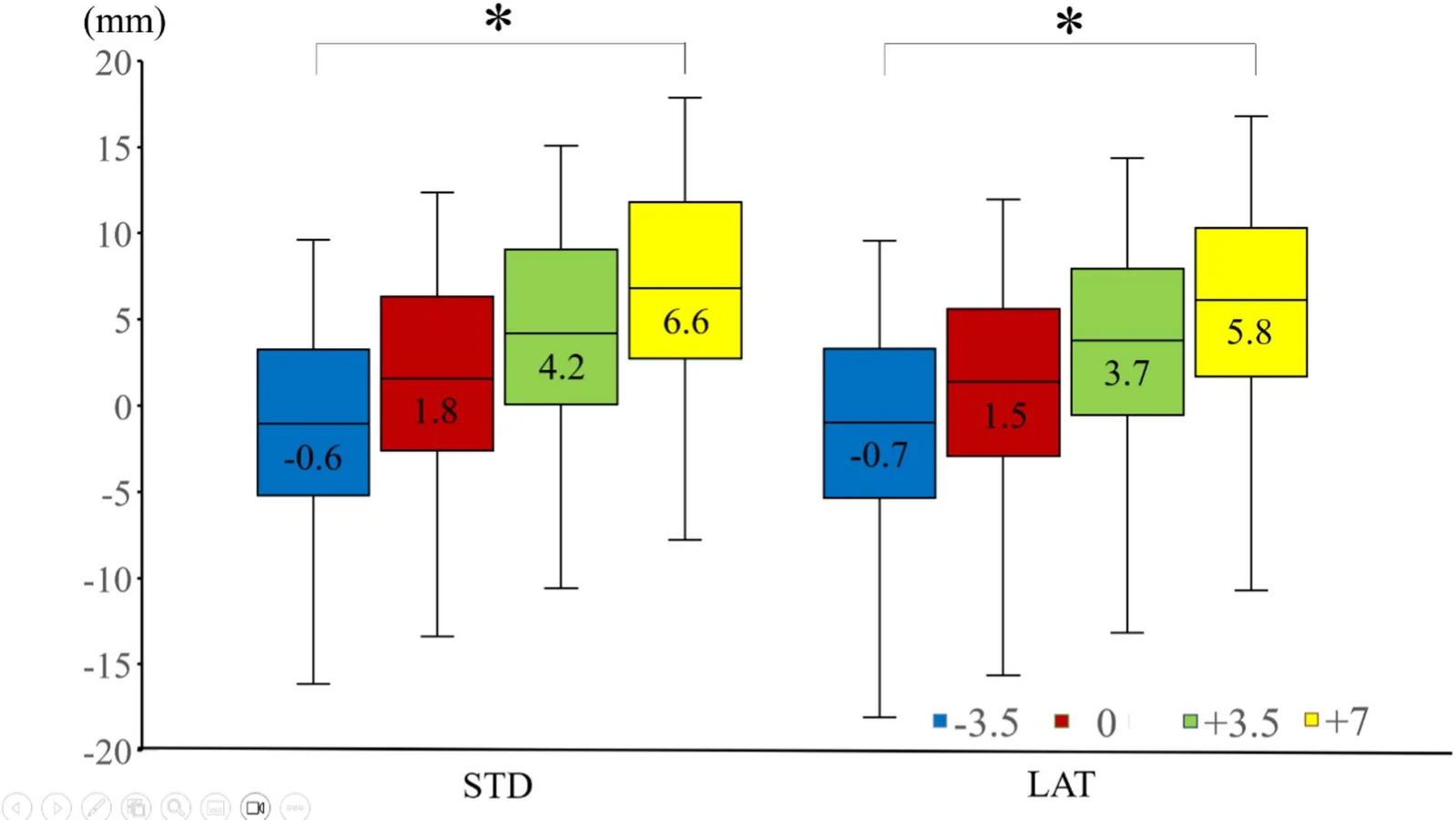
Effect of neck length and offset on simulated leg length discrepancy. In Group A, leg length discrepancy relative to the contralateral healthy hip increased with each 3.5-mm increase in neck length in both STD and LAT stems (*p* = 0.001 for each). No substantial difference in leg length discrepancy was observed between the two stem configurations at the same neck length

Simulated LLD relative to the contralateral healthy hip increased by a median of 2.3 mm (range, 2.1–2.4 mm) for every 3.5-mm increase in neck length (Fig. 8). No substantial difference in LLD was observed between STD and LAT stems at the same neck length. Median LLD remained within 5 mm for STD stems at − 3.5 mm, 0 mm, and + 3.5 mm, and for LAT stems at − 3.5 mm and 0 mm.

**Fig. 9:**
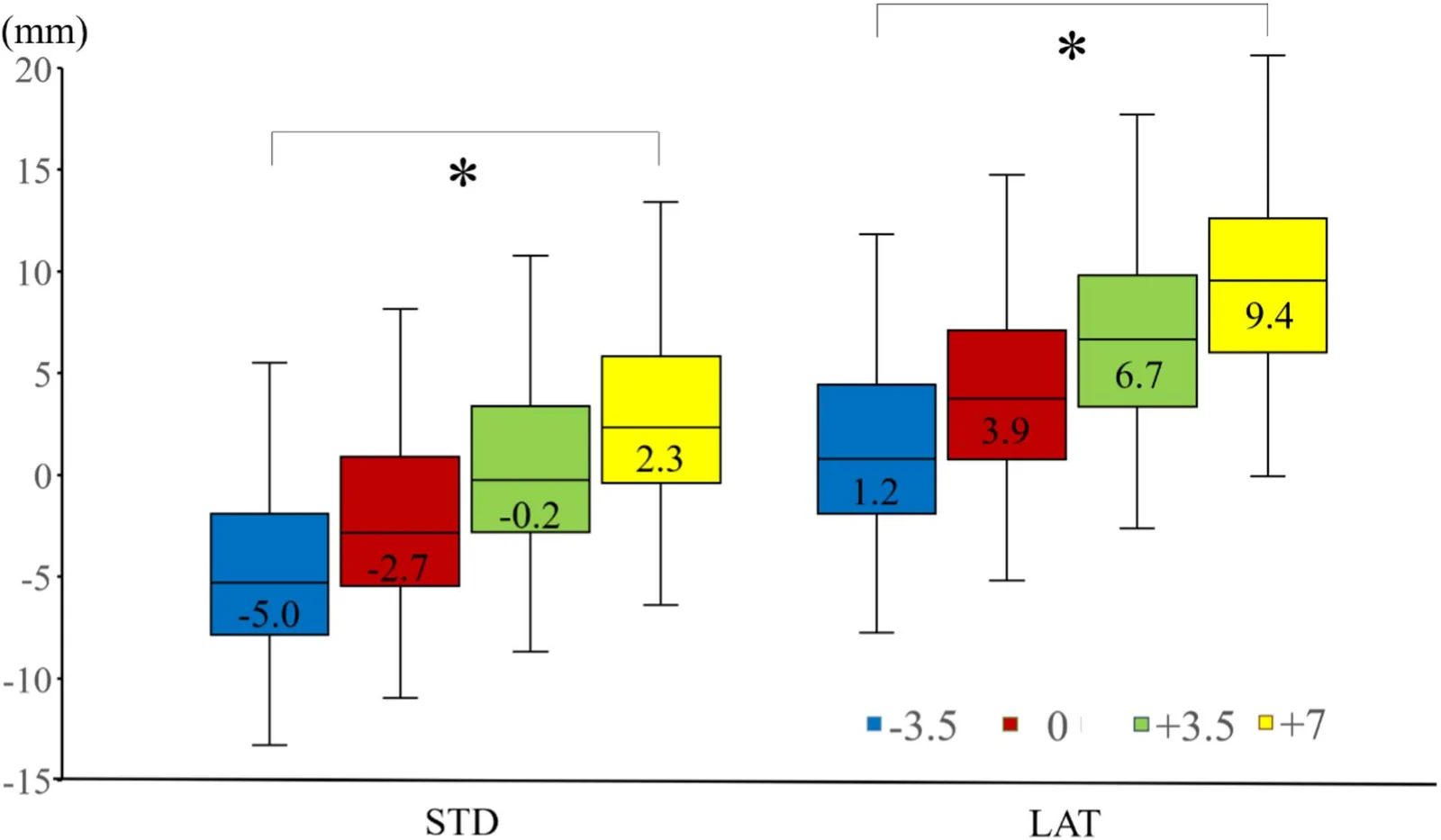
Effect of neck length and offset on simulated femoral offset discrepancy. In Group A, the offset discrepancy relative to the contralateral healthy hip increased with each 3.5-mm increase in neck length in both STD and LAT stems (*p* = 0.001 for each). At the same neck length, femoral offset discrepancy was greater with the LAT stem than with the STD stem (p = 0.001 for each comparison)

Simulated femoral offset discrepancy relative to the contralateral healthy hip increased by a median of 2.6 mm (range, 2.3–2.8 mm) for every 3.5-mm increase in neck length (Fig. 9). At the same neck length, LAT stems increased femoral offset discrepancy by a median of 6.8 mm (range, 6.6–7.1 mm) compared with STD stems. Median femoral offset discrepancy remained within 5 mm for STD stems from − 3.5 mm to + 7 mm and for LAT stems at − 3.5 mm and 0 mm.

### Effect of femoral head diameter

Comparison between Groups A and B showed that reducing the femoral head diameter from 36 mm to 32 mm resulted in a consistent decrease in simulated impingement-free mechanical ROM across implant configurations. Maximum flexion decreased by a median of 2.5° (range, 1.5°–4.5°) (Fig. 4; Table 2). Maximum extension decreased by a median of 2.5° (range, 1.0°–8.5°) (Fig. 5; Table 2). Similarly, maximum internal rotation decreased by a median of 2.8° (range, 0.5°–4.5°) (Fig. 6; Table 2), and maximum external rotation decreased by a median of 2.0° (range, 1.0°–4.0°) (Fig. 7; Table 2).

**Table 2:** Simulated mechanical ROM, leg length discrepancy, and femoral offset discrepancy for cups ≥ 50 mm with a 32-mm head (Group B)

Stem type	STD				LAT				Healthy side
Neck length	−3.5 mm	0 mm	+ 3.5 mm	+ 7 mm	−3.5 mm	0 mm	+ 3.5 mm	+ 7 mm	
Maximum flexion	88.5 ± 19.6	97.0 ± 19.1	106.0 ± 18.0	111.5 ± 15.5	105.0 ± 20.6	115.0 ± 18.9	121.5 ± 17.7	127.0 ± 13.5	119.0 ± 13.3
Maximum extension	37.0 ± 17.3	52.0 ± 15.9	64.0 ± 11.9	62.5 ± 9.7	58.5 ± 13.2	66.5 ± 13.1	69.0 ± 12.2	64.0 ± 11.0	66.0 ± 19.2
Maximum internal rotation	18.0 ± 17.9	29.5 ± 10.4	36.0 ± 10.3	35.0 ± 8.4	35.0 ± 10.3	38.5 ± 10.1	40.5 ± 8.8	38.5 ± 9.1	31.0 ± 15.1
Maximum external rotation	32.5 ± 9.8	39.5 ± 10.5	44.5 ± 11.4	50.5 ± 10.2	36.5 ± 9.4	40.0 ± 10.2	44.0 ± 10.2	47.5 ± 8.7	48.0 ± 13.1
Leg length discrepancy	-1.2 ± 6.3	1.2 ± 6.3	3.6 ± 6.3	6.0 ± 6.3	-1.0 ± 6.3	1.2 ± 6.4	3.3 ± 6.3	5.5 ± 6.3	**-**
Femoral offset discrepancy	-5.0 ± 4.5	-2.7 ± 4.5	-0.2 ± 4.6	2.3 ± 4.6	1.2 ± 4.6	3.9 ± 4.7	6.7 ± 4.7	9.4 ± 4.7	**-**

Mean ± standard deviation. The multiple comparisons within the STD group, ranging from − 3.5 mm to + 7 mm, show significant differences in each ROM and length (*p* = 0.001). The multiple comparisons within the LAT group, ranging from − 3.5 mm to + 7 mm, also show significant differences in each ROM and length (*p* = 0.001)

### Effect of acetabular cup diameter

Comparisons between Groups B and C showed direction-dependent differences in simulated impingement-free mechanical ROM. Compared with Group B, Group C showed greater maximum flexion by a median of 4.8° (range, 2.5°–7.0°) and greater maximum internal rotation by a median of 5.8° (range, 3.0°–8.5°) (Tables 2 and 3). In contrast, maximum extension was lower in Group C by a median of 9.0° (range, 3.5°–15.5°). No significant difference in maximum external rotation was observed between the two groups. In addition, the median stem size was 4 (range, 1–8) in Groups B and 2 (range, 01–5) in Group C (Table 1). Stem size was significantly smaller in Group C than in Groups B (*p* < 0.001). The median increase in femoral offset was 7.0 mm in Groups B and 6.5 mm in Group C, and this difference was significant (*p* < 0.001).

**Table 3:** Simulated mechanical ROM, leg length discrepancy, and femoral offset discrepancy for cups < 50 mm with a 32-mm head (Group C)

Stem type	STD				LAT				Healthy side
Neck length	-3.5 mm	0 mm	+ 3.5 mm	+ 7 mm	-3.5 mm	0 mm	+ 3.5 mm	+ 7 mm	
Maximum flexion	93.5 ± 16.6	101.5 ± 15.1	127.5 ± 11.8	116.0 ± 9.7	112.0 ± 14.9	121.0 ± 11.4	127.0 ± 10.6	129.5 ± 7.8	127.5 ± 14.6
Maximum extension	32.0 ± 16.2	48.5 ± 16.1	54.5 ± 11.9	54.5 ± 9.7	48.0 ± 15.1	51.0 ± 13.4	55.0 ± 12.5	55.5 ± 11.7	69.0 ± 25.6
Maximum internal rotation	25.0 ± 13.6	34.0 ± 11.6	39.0 ± 10.0	44.0 ± 7.7	38.0 ± 10.4	43.0 ± 8.9	49.0 ± 7.9	47.0 ± 7.1	49.0 ± 16.2
Maximum external rotation	32.0 ± 9.7	40.0 ± 10.0	45.0 ± 10.0	48.0 ± 9.4	35.0 ± 9.9	41.0 ± 9.3	44.0 ± 9.2	43.0 ± 8.2	44.0 ± 14.3
Leg length discrepancy	0.3 ± 5.6	2.8 ± 5.6	5.4 ± 5.6	8.0 ± 5.6	0.8 ± 5.6	3.0 ± 5.6	5.2 ± 5.6	7.5 ± 5.6	**-**
Femoral offset discrepancy	-3.1 ± 3.7	-0.7 ± 3.7	1.5 ± 3.7	3.7 ± 3.7	2.5 ± 3.8	5.0 ± 3.8	7.5 ± 3.9	10.2 ± 3.9	**-**

Mean ± standard deviation. Mean ± standard deviation. The multiple comparisons within the STD group, ranging from − 3.5 mm to + 7 mm, show significant differences in each ROM and length (*p* = 0.001). The multiple comparisons within the LAT group, ranging from − 3.5 mm to + 7 mm, also show significant differences in each ROM and length (*p* = 0.001)

### Simulated ROM of the contralateral healthy hip

The contralateral healthy hip was evaluated as a patient-specific comparator. The median simulated ROM of the contralateral healthy hips was 119.0° (range, 80°–144°) for maximum flexion, 66.0° (range, 20°–106°) for maximum extension, 31.0° (range, 13°–49°) for internal rotation at 90° of flexion, and 48.0° (range, 5°–59°) for external rotation at 10° of extension (Figs. 4, 5, 6 and 7).

## Discussion

This CT-based three-dimensional simulation study investigated the effects of femoral head diameter, neck length, and stem offset on simulated impingement-free mechanical ROM, LLD, and femoral offset discrepancy after unilateral THA. The main findings were as follows: first, increasing neck length and femoral offset increased simulated mechanical ROM in all evaluated directions; second, femoral head enlargement provided a modest additional improvement in simulated mechanical ROM; third, neck lengthening improved mechanical ROM but simultaneously increased LLD and femoral offset discrepancy; and fourth, the contralateral healthy hip showed a median simulated flexion of 119°, with a wide individual range from 80° to 144°. These findings suggest that implant-related parameters have measurable effects on mechanical hip motion and that the contralateral healthy hip may provide a useful patient-specific comparator in unilateral THA.

Normal hip ROM has been reported with considerable variability in the literature. Using maximum hip flexion as an example, previous studies have reported values ranging from approximately 96° to 141° in asymptomatic or healthy subjects [5–10]. This variability is partly explained by differences in age, sex, active versus passive measurement, pelvic stabilization, and whether terminal motion is defined clinically or by imaging-confirmed intra-articular events. Svenningsen et al. reported adult passive flexion of 141° in females and 137° in males, while also showing that hip motion decreases with age and differs by sex [5]. Roaas and Andersson reported hip flexion of approximately 120° in healthy male subjects aged 30–40 years, and other clinical studies have also reported flexion values around 120° [6–9]. In contrast, Larkin et al. demonstrated that maximum midsagittal passive flexion at bony abutment was approximately 96° in asymptomatic young adult males using dynamic ultrasound, suggesting that conventional clinical measurements may include pelvic or lumbosacral motion and may overestimate isolated intra-articular hip motion [10]. Thus, normal hip ROM should not be regarded as a fixed universal value, but rather as a measurement-dependent and patient-dependent parameter.

In the present study, the median simulated flexion of the contralateral healthy hip was 119°. This value is consistent with commonly cited clinical values of normal hip flexion of approximately 120° and lies within the range reported in previous studies. Therefore, the simulated flexion of the contralateral healthy hip in this cohort appears reasonable as a representative value for mechanical hip motion. At the same time, the range of simulated flexion among the contralateral healthy hips was broad, from 80° to 144°. This wide distribution indicates that hip ROM differs substantially among individuals, even when the hip is clinically and radiographically considered healthy. Therefore, interpretation of simulated postoperative mechanical ROM after THA should not rely solely on population-based normative values.

The ROM of the contralateral healthy hip may provide a patient-specific comparator when interpreting implant configurations during preoperative planning. This strategy allows each patient to serve as their own control, thereby minimizing interindividual variability in sex, body height, pelvic morphology, and spinopelvic characteristics that may confound comparisons across different subjects. Previous studies have suggested that the contralateral side can provide a patient-specific benchmark for joint motion and implant positioning. For example, Nakanishi et al. [26] demonstrated that the mean flexion angle of the contralateral healthy hip under anesthesia was 109.4° ± 6.9° (range, 88°–126°) in 83 consecutive patients undergoing primary unilateral THA. Furthermore, Palit et al. [27] proposed that the impingement-free ROM of the contralateral healthy hip may serve as a subject-specific reference when determining the achievable impingement-free ROM on the operative side. By comparing simulated implant scenarios with each individual’s contralateral hip ROM, the present approach may provide a more individualized context for interpreting mechanical ROM than reliance on population-based normative values alone. However, the contralateral hip should be regarded as a patient-specific comparator rather than a validated physiological target.

Femoral head enlargement improved simulated mechanical ROM; however, its effect was limited compared with the effects of neck length and stem offset. Reducing the femoral head diameter from 36 mm to 32 mm produced a consistent but small decrease in simulated mechanical ROM across implant configurations. This finding is consistent with previous experimental and simulation studies showing that larger femoral heads can improve stability and ROM [33]. Increasing femoral head diameter may improve prosthetic clearance and jumping distance, whereas increasing offset may delay osseous or implant-related impingement between the femur, pelvis, and prosthetic components. Therefore, although femoral head enlargement improved simulated mechanical ROM, its clinical relevance should be interpreted together with other reconstructive parameters, including offset, leg length, cup orientation, and stem version.

This study has several limitations. First, the ROM analyzed in this study represents simulated impingement-free mechanical ROM and does not necessarily correspond to functional ROM achievable in vivo. Clinical hip motion is influenced by osteophyte resection to reduce bony impingement, femoral neck cut position to reduce LLD, soft tissue tension, capsular restraint, muscle stiffness, pain, patient behavior, and pelvic or lumbar motion. Furthermore, although the endpoint of simulated ROM was determined by the onset of impingement, the type of impingement was not systematically classified as bone-to-bone, implant-to-bone, or implant-to-implant impingement. Additionally, in external rotation at 10° of extension, the LAT stem increased ROM less than expected compared with the STD stem at the same neck length. One possible explanation is that this direction of motion may have a different impingement mechanism from flexion or internal rotation because of the complex anatomy of the greater trochanter, femoral neck, and posterior bony structures of the acetabulum. Therefore, the simulated ROM values should be interpreted as theoretical mechanical limits under standardized conditions, not as actual postoperative ROM. Second, the present analysis was performed using a single implant system. Implant geometry, including neck design, taper geometry, head–neck ratio, offset increments, and the relationship between neck length and femoral head center, varies among manufacturers. Therefore, the numerical values observed in this study may not be directly generalizable to other implant systems. Third, interpretation of the comparison between Groups B and C requires caution because several anatomical variables differed simultaneously between the groups, including sex distribution, body size, canal morphology, and femoral anteversion. Specifically, Group C consisted entirely of female patients and had shorter height, lower body weight, greater stem anteversion, and narrower medullary canals than Group B. These factors may independently influence impingement patterns and simulated ROM. Therefore, the observed differences between Groups B and C cannot be attributed solely to cup diameter. This comparison should be interpreted as reflecting both cup diameter and anatomical differences between the groups. Fourth, cup alignment was fixed at 40° inclination and 15° anteversion according to Lewinnek’s safe zone. However, in contemporary THA practice, component orientation is often individualized according to combined anteversion, spinopelvic dynamics, and acetabular bone coverage. In addition, some deviation from the planned position is inevitable during surgery, and even small changes in component orientation may affect the onset of impingement. Finally, although the contralateral hip was used as a patient-specific comparator, it may not always represent a completely normal joint in patients with unilateral hip disease. Subclinical morphology, compensatory loading, or early degenerative changes may exist even in an asymptomatic contralateral hip. Especially, in patients with developmental dysplasia of the hip, pelvic asymmetry and the influence of postoperative pelvic tilt should be considered.

## Conclusions

This CT-based three-dimensional simulation study showed that increasing neck length and lateral offset improved simulated impingement-free mechanical ROM after unilateral THA, whereas neck lengthening increased LLD. The median simulated flexion of the contralateral healthy hip was 119°, consistent with commonly reported normal hip flexion values, but its wide range suggested substantial interindividual variability. The contralateral healthy hip may provide contextual patient-specific information for interpreting simulated mechanical ROM in unilateral THA. 

## Data Availability

The datasets generated and/or analyzed during the current study are not publicly available because they contain patient imaging data. Anonymized data may be available from the corresponding author upon reasonable request and with permission from the Institutional Review Board.

## Abbreviations

3D: Three-dimensional
BMI: Body mass index
CT: Computed tomography
LAT: Lateral offset
LLD: Leg length discrepancy
MRI: Magnetic resonance imaging
OA: Osteoarthritis
ONFH: Idiopathic osteonecrosis of the femoral head
RA: Rheumatoid arthritis
ROM: Range of motion
STD: Standard
THA: Total hip arthroplasty
ZTA: Zirconia-toughened alumina
